# *Angiopoietin-2* Is a Direct Transcriptional Target of TAL1, LYL1 and LMO2 in Endothelial Cells

**DOI:** 10.1371/journal.pone.0040484

**Published:** 2012-07-06

**Authors:** Virginie Deleuze, Rawan El-Hajj, Elias Chalhoub, Christiane Dohet, Valérie Pinet, Philippe Couttet, Danièle Mathieu

**Affiliations:** 1 Institut de Génétique Moléculaire de Montpellier UMR 5535 CNRS, Montpellier, France; 2 Université Montpellier 2, Montpellier, France; 3 Université Montpellier 1, Montpellier, France; 4 The Sanger Institute, Cambridge, United Kingdom; University of Illinois College of Medicine, United States of America

## Abstract

The two related basic helix–loop-helix, TAL1 and LYL1, and their cofactor LIM-only-2 protein (LMO2) are present in blood and endothelial cells. While their crucial role in early hematopoiesis is well established, their function in endothelial cells and especially in angiogenesis is less understood. Here, we identified *ANGIOPOIETIN-2 (ANG-2)*, which encodes a major regulator of angiogenesis, as a direct transcriptional target of TAL1, LYL1 and LMO2. Knockdown of any of the three transcription factors in human blood and lymphatic endothelial cells caused *ANG-2* mRNA and protein down-regulation. Transient transfections showed that the full activity of the *ANG-2* promoter required the integrity of a highly conserved Ebox-GATA composite element. Accordingly, chromatin immunoprecipitation assays demonstrated that TAL1, LYL1, LMO2 and GATA2 occupied this region of *ANG-2* promoter in human endothelial cells. Furthermore, we showed that LMO2 played a central role in assembling TAL1-E47, LYL1-LYL1 or/and LYL1-TAL1 dimers with GATA2. The resulting complexes were able to activate endogenous *ANG-2* expression in endothelial cells as well as in non-endothelial cells. Finally, we showed that *ANG-2* gene activation during angiogenesis concurred with the up-regulation of TAL1 and LMO2. Altogether, we identified *ANG-2* as a *bona fide* target gene of LMO2-complexes with TAL1 and/or LYL1, highlighting a new function of the three hematopoietic factors in the endothelial lineage.

## Introduction

Angiogenesis, the process by which endothelial cells (ECs) form new blood vessels from an existing vascular network, is critical in embryonic development and in a process like wound healing in adults. Angiogenesis also contributes to inflammation diseases and tumor development, while insufficient angiogenesis leads to ischemia. Angiogenesis requires multiple cellular processes, including migration, proliferation, morphogenesis and cell-cell communication (see review [1]). Hence, investigating the transcriptional mechanisms controlling and coordinating this complex process represents a major aspect in vascular biology.

Hematopoietic and endothelial cells are intimately associated throughout both embryonic and adult life, and recent studies have identified that hematopoietic stem cells (HSCs) have an endothelial origin [2]–[4]. Given their common origin, blood and endothelial cells share multiple transcription factors regulating their development and their differentiation. Among these, are two related members of the basic helix–loop-helix (bHLH) family, TAL1/SCL and LYL1, and the LIM-only-2 protein (LMO2).

During development, *Tal1*, *Lyl1* and *Lmo2* display wide overlapping expression in both immature hematopoietic cells and in endothelium [5], [6]. Genetic studies in mice have determined the key roles of *Tal1* or *Lmo2* in the formation of HSCs [6], [7] and in the development of the vascular system [8]–[12]. The virtually identical phenotypes in blood and endothelium of *Tal1-* and *Lmo2*-deficient embryos have been attributed to the fact that the two proteins function together as key components of multiprotein-DNA complexes to control hematopoietic-specific (reviewed in [7]) and endothelial-specific genes [13], [14]. Accordingly, a non-LMO2 binding form of TAL1 cannot rescue *Tal1*-deficiency [15].

Unlike *Tal1*, *Lyl1* is dispensable for embryonic development [16], presumably because *Tal1* compensates for the lack of *Lyl1* during development. However, adult mice lacking *Lyl1* mice have reduced numbers of repopulating HSCs and mature B cells [16]. Consistent with their redundant function in adult HSCs [17], several genome-wide analyses have revealed that TAL1, LMO2 and LYL1 function with other hematopoietic-specific transcription factors in high-order complexes to regulate transcriptional programs responsible for the maintenance and differentiation of HSCs [18]–[21].

*Tal1* and *Lyl1* exhibit differences in adult tissues. *Tal1* expression, undetectable in quiescent mature endothelium, occurs in forming vessels [22], [23], including vascular proliferations and tumor lymphatic vessels [24]–[26]. Accordingly, we identified that TAL1 acts as a positive factor for postnatal angiogenesis [27], and particularly during endothelial morphogenesis where, conjointly with LMO2 and GATA2, it activates *VE-cadherin* encoding the major constituent of endothelial adherens junctions [13].

**Figure 1:**
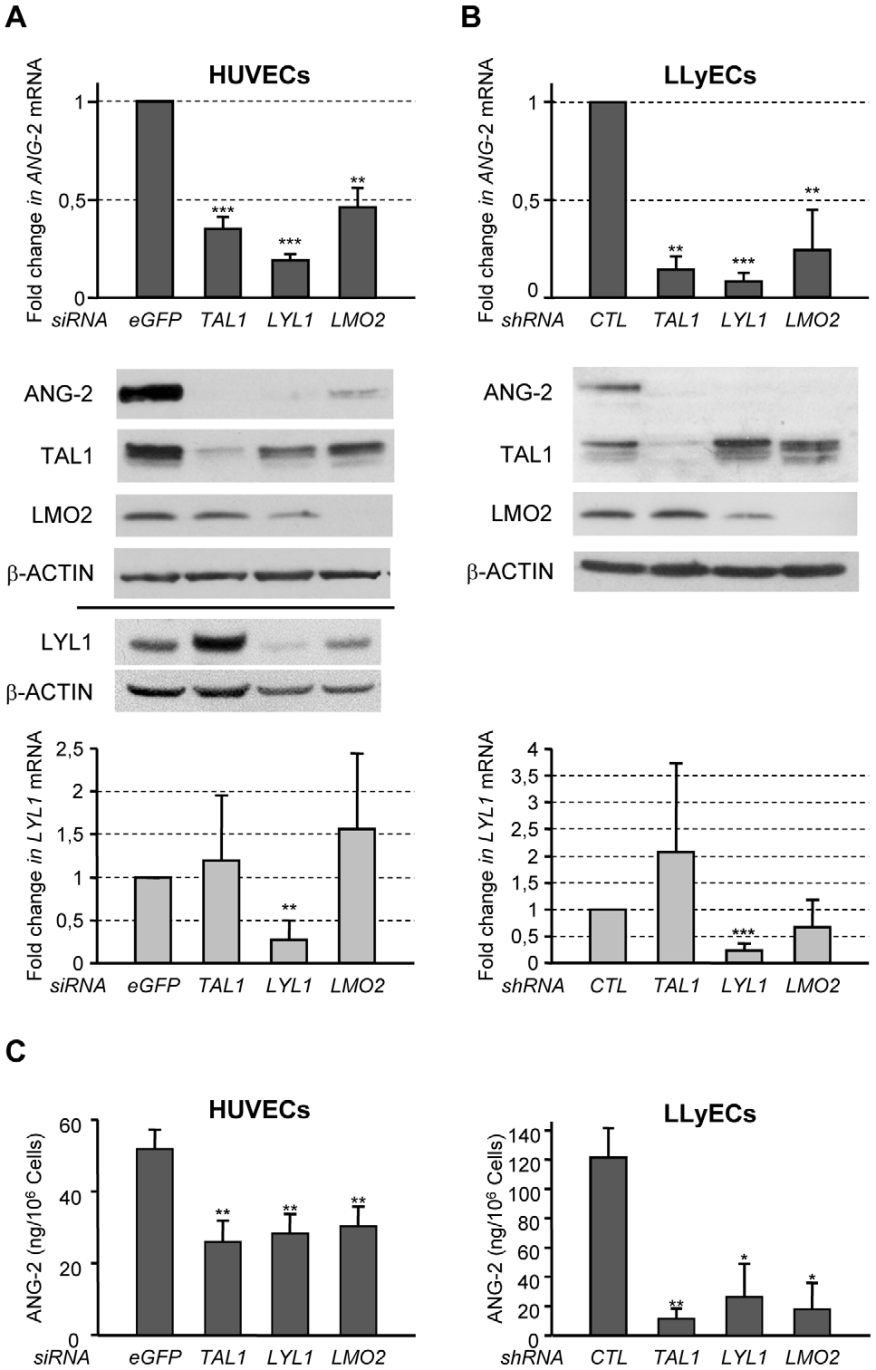
TAL1-, LYL1- or LMO2-silencing down regulates *ANG-2* expression. (**A, C**) HUVECs were transfected with siRNAs targeting *TAL1*, *LYL1*, *LMO2* or *eGFP* (used as the control). Whole cell extracts and total RNAs were prepared 48 hours post transfection for expression analysis. (**B, C**) LLyECs were transduced with pLKO.1 lentiviruses encoding shRNA targeting *TAL1*, *LYL1*, *LMO2* or control shRNA. Puromycine-resistant populations were analyzed between 12 to 20 days after transduction. (**A. B**) Top, bottom *ANG-2* and *LYL1* mRNA levels was analyzed by RT-q-PCR. The signals of *ANG-2* and *LYL1* mRNA were normalized to those of *GAPDH*. The data are shown relative to mRNA content from control siRNA-treated cells arbitrarily set at 1. Each bar is the mean +/− SD of three independent experiments. Middle Intracellular ANG-2, TAL1, LMO2 and LYL1 protein expression was analyzed by immunoblotting. β-ACTIN expression was monitored to control protein loading (the bar indicates two independent immunoblots). The images shown are representative of three independent experiments. **, *P*<0.01; ***, *P*<0.001. (**C**) TAL1-, LYL1- or LMO2-depletion reduces constitutive ANG-2 secretion in culture medium. Left: HUVECs were transfected with the indicated siRNA. 24 hours after transfection, medium was changed and ANG2 secretion into culture medium for 24 hours was measured by ELISA. Right: LLyECs were transduced with pLKO.1 lentiviruses encoding the indicated shRNA and puromycine-resistant cell populations were grown to confluence. ELISA was used to measure ANG2 release for 48 hours into the media. ANG-2 production is shown relative to cell number measured at the end of the culture. *, *P*<0.05; **, *P*<0.01; ***, *P*<0.001.

Unlike *Tal1*, *Lmo2* and *Lyl1* are expressed in growing vessels but also in resting endothelium [26], [28], [29]. We reported that adult *Lyl1*–deficient mice, although they do not display obvious vascular abnormalities, exhibit increased angiogenesis in hypoxic and non-hypoxic conditions [26]. We highlighted this phenotype by identifying that LYL1 controls the expression of endothelial molecules involved in the maturation of vascular structures [26].

**Figure 2:**
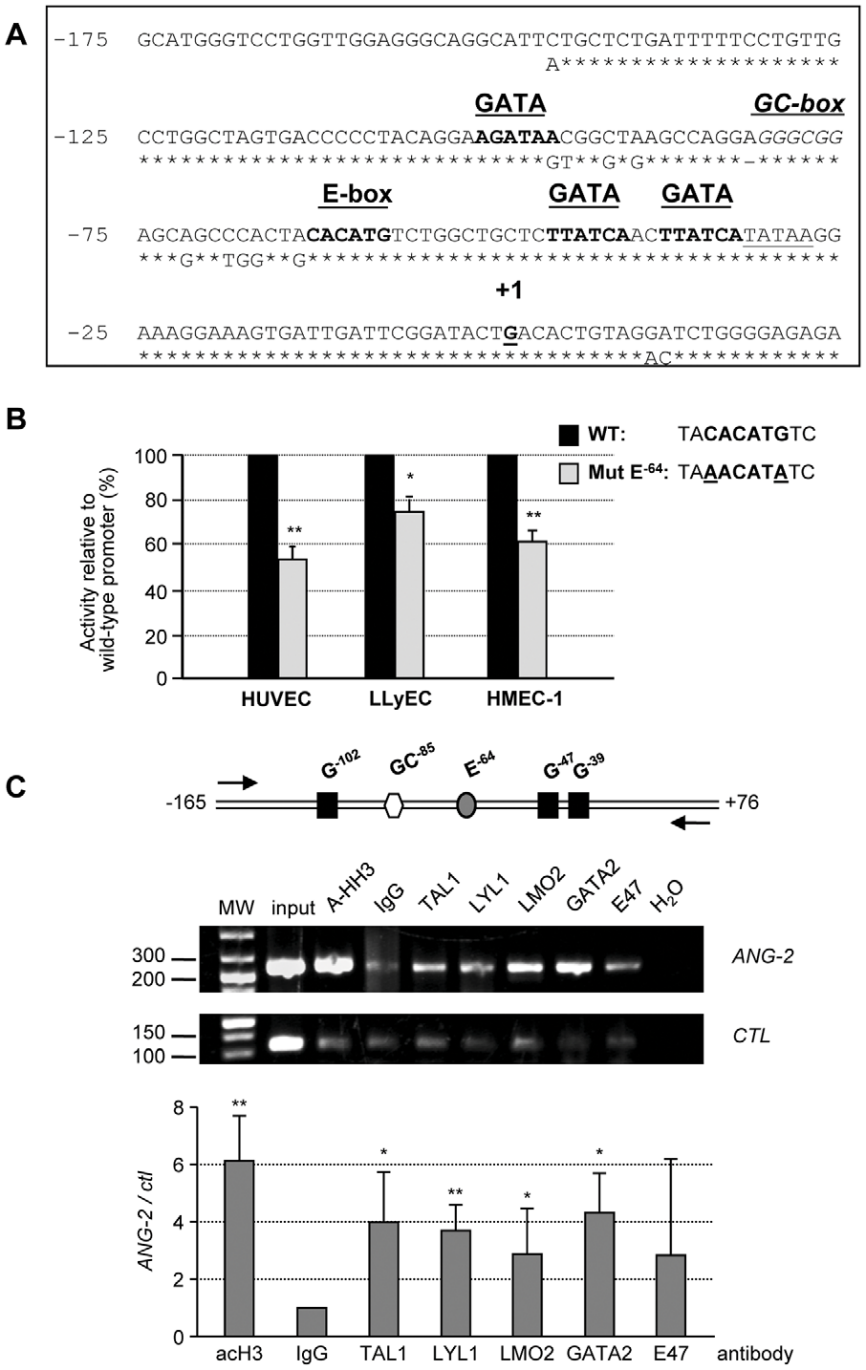
TAL1, LYL1, LMO2 and GATA2 bind the *ANG-2* promoter proximal region. (**A**) Alignment of the nucleotide sequence of proximal region of human (top line) and mouse *ANG-2* promoter. The conserved E-box, GATA-binding sites and GC-box boxes are indicated. The TATA box is underlined. +1 indicates the transcription start site. Of note, the E box^−64^ is separated by 11 nucleotides from both the upstream GC-box^−85^ and the downstream GATA^−47^. (**B**) The indicated endothelial cells were transfected with either the wild-type −412/+73 *ANG-2* reporter construct (black bars) or the mutated Ebox^−64^ construct (grey bars). Data are shown relative to the luciferase activity of wt construct (set at 100%) for each cell type. Bars are the means +/− S.D. of three independent experiments, each performed in triplicate. **, *P*<0.01; *, *P*<0.05. (**C**) Chromatin immunoprecipitation assays. Chromatin immunoprecipitations assays (ChIP) were performed on cross-linked chromatin from LLyECs using the indicated antibody. To control chromatin quality, ChIP assays were performed using an antibody against acetylated histone H3. Aliquots of immunoprecipitated DNAs were amplified by PCR with primers (arrows) targeting the proximal *ANG-2* promoter (−165; +76) or a non-transcribed genomic region upstream of the *c-KIT* gene used as a negative control. PCR products were analyzed by gel electrophoresis. Top: Schematic location of conserved elements within the human *ANG-2* promoter: G: GATA-binding site; GC: GC-box; E: E-box. Shown images are representative of one of three independent experiments. Bottom: Fold-enrichment of target genomic regions immunoprecipitated by each antibody was normalized to the levels obtained with control IgGs, which was assigned a value of 1, and plotted as the increase over the levels of enrichment at the negative control region. The error bars represent +/− S.D. from three independent ChIPs. **, *P*<0.01; *, *P*<0.05.

Given the concomitant expression of TAL1, LYL1 and LMO2 in angiogenic endothelium and since the 3 factors function together in DNA-multiprotein complexes in hematopoietic cells [21], [30], we speculated that they might also cooperate in ECs to control angiogenic processes. In this study, we set out to identify endothelial-specific target genes common to the 3 factors and having a role during angiogenesis. The *Angiopoietin-2* (*ANG-2*) gene emerged as a common transcriptional target downstream of TAL1, LYL1 and LMO2. We provide evidence that several multi-protein complexes including TAL1 and/or LYL1, LMO2 and GATA2 up-regulate *ANG-2* expression through direct binding to the *ANG-2* proximal promoter.

**Figure 3:**
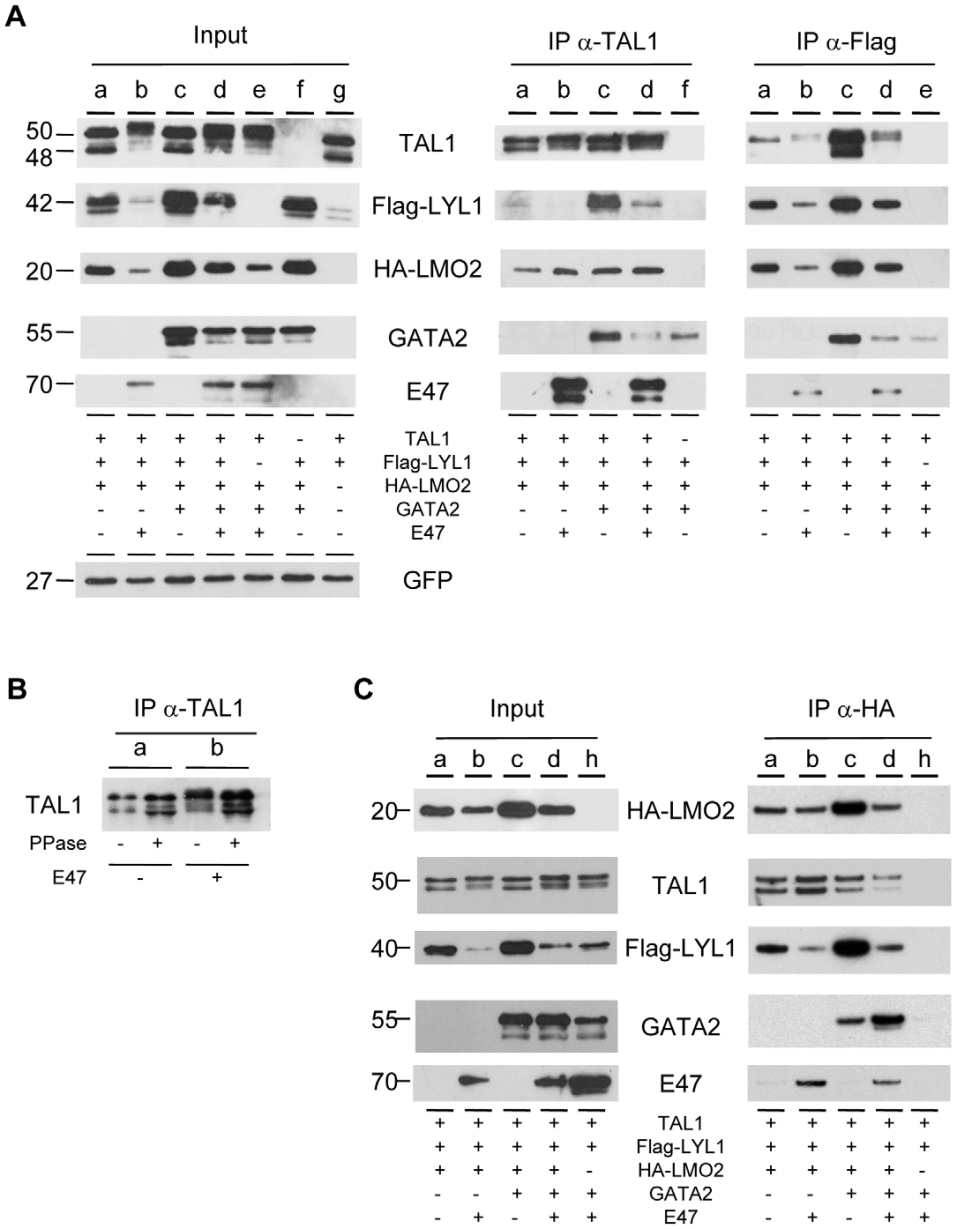
Analysis of TAL1, LYL1 and LMO2 interactions in cells. 293T cells were cotransfected with the indicated expression vectors along with eGFP-expressing vector to control transfection efficacy. (**A**) TAL1 and LYL1 interactions: Equal amounts of whole cell lysates (WCL) were immunoprecipitated with the indicated antibody. Mouse α-Flag- mAb was used to precipitate Flag-LYL1. Input shows levels of tested proteins in untreated WCL (10 µg per lane). Input and immunoprecipitated proteins were analyzed by immunoblot using the appropriate antibodies. (**B**) E47 induces TAL1 hyperphosphorylation: WCL from cells transfected with combination a or b (indicated in A) were immunoprecipitated with mouse α-TAL1 mAb. Immunoprecipitates coupled to sepharose were treated by Calf Intestinal alcaline Phosphatase (CIP) and analyzed by immunoblot using goat α-TAL1 pAb. (**C**) LMO2 interactions: Equal amounts of WCL were treated with mouse α-HA-mAb coupled to agarose to precipitate HA-LMO2. Input shows levels of tested proteins in untreated WCL (10 µg per lane). Input and immunoprecipitated proteins were analyzed by immunoblot using the appropriate antibodies.

## Materials and Methods

### Cell cultures

HEK-293T cells, obtained from ATTCC, were grown in Dulbecco's modified Eagle's medium (DMEM) supplemented with 10% FBS and antibiotics. Primary human endothelial cells (ECs) from umbilical vein (HUVECs) and Lung Lymphatic Microvascular Endothelial Cells (LLyECs) were obtained respectively from PromoCell GmbH (Heidelberg, Germany) and Clonetics (LONZA, Belgium). The human cell line HMEC-1 [31] was provided by the Center for Disease Control (CDC, Atlanta, USA). All ECs were cultured in complete endothelial cell growth medium MV2 (PromoCell GmbH, Heidelberg, Germany).

**Figure 4:**
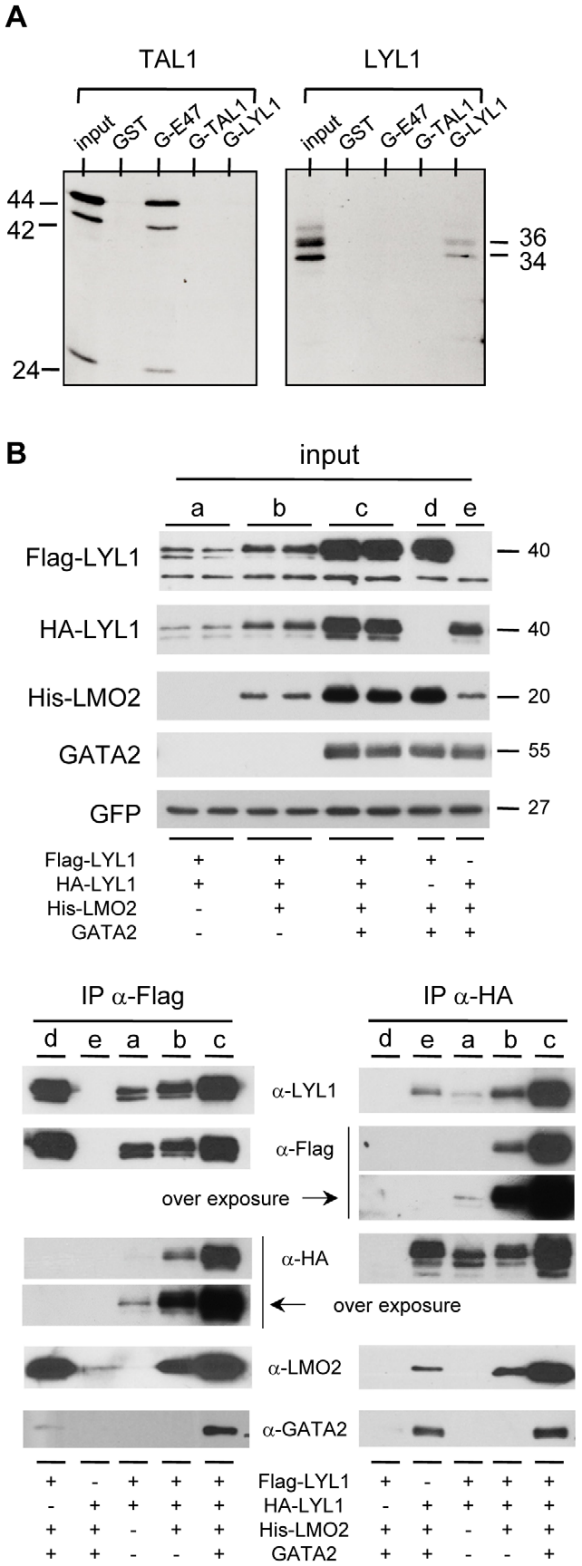
LYL1 is able to form homodimers *in vitro* and *in vivo*. (**A**) GST pull-down assays: ^35^S-labeled TAL1 and LYL1 proteins produced *in vitro* by coupled transcription-translation were incubated with the indicated GST protein coupled to glutathione-Sepharose beads. Captured radiolabeled proteins were analyzed by SDS-PAGE and visualized by autoradiography. (**B**) LYL1 forms homodimers in cells: 293T cells were transfected with Flag-LYL1 and/or HA-LYL1. Equal amounts of WCL were immunoprecipitated with α-Flag- and α-HA-antibodies to precipitate LYL1-Flag and LYL1-HA respectively. Input shows levels of tested proteins in untreated WCL (10 µg per lane). Input and immunoprecipitated proteins were analyzed by immunoblot using the indicated antibody.

### siRNA transfections

Small interfering RNAs (siRNA) were transfected in HUVECs as described [13]. The sequences of duplex RNAs were as follows:

*eGFP*-Forward CUACAACAGCCACAACGUC-TT

*eGFP*-Reverse GACGUUGUGGCUGUUGUAG-TT

*TAL1*-Forward GAAGCUCAGCAAGAAUGAG-TT

*TAL1*-Reverse CUCAUUCUUGCUGAGCUUC-TT

*LYL1*-Forward GAUGGAGCAAACCGCUUUG-TT

*LYL1*-Reverse CAAAGCGGUUUGCUCCAUC-TT

*LMO2-A*-Forward GCAUCCAAGUGGCAUAAUU-TT

*LMO2-A*-Reverse AAUUAUGCCACUUGGAUGC-TT

*LMO2-B*-Forward CUAGAGAUGUGCAAUUGAU-TT

*LMO2-B*-Reverse AUCAAUUGCACAUCUCUAG-TT

**Figure 5:**
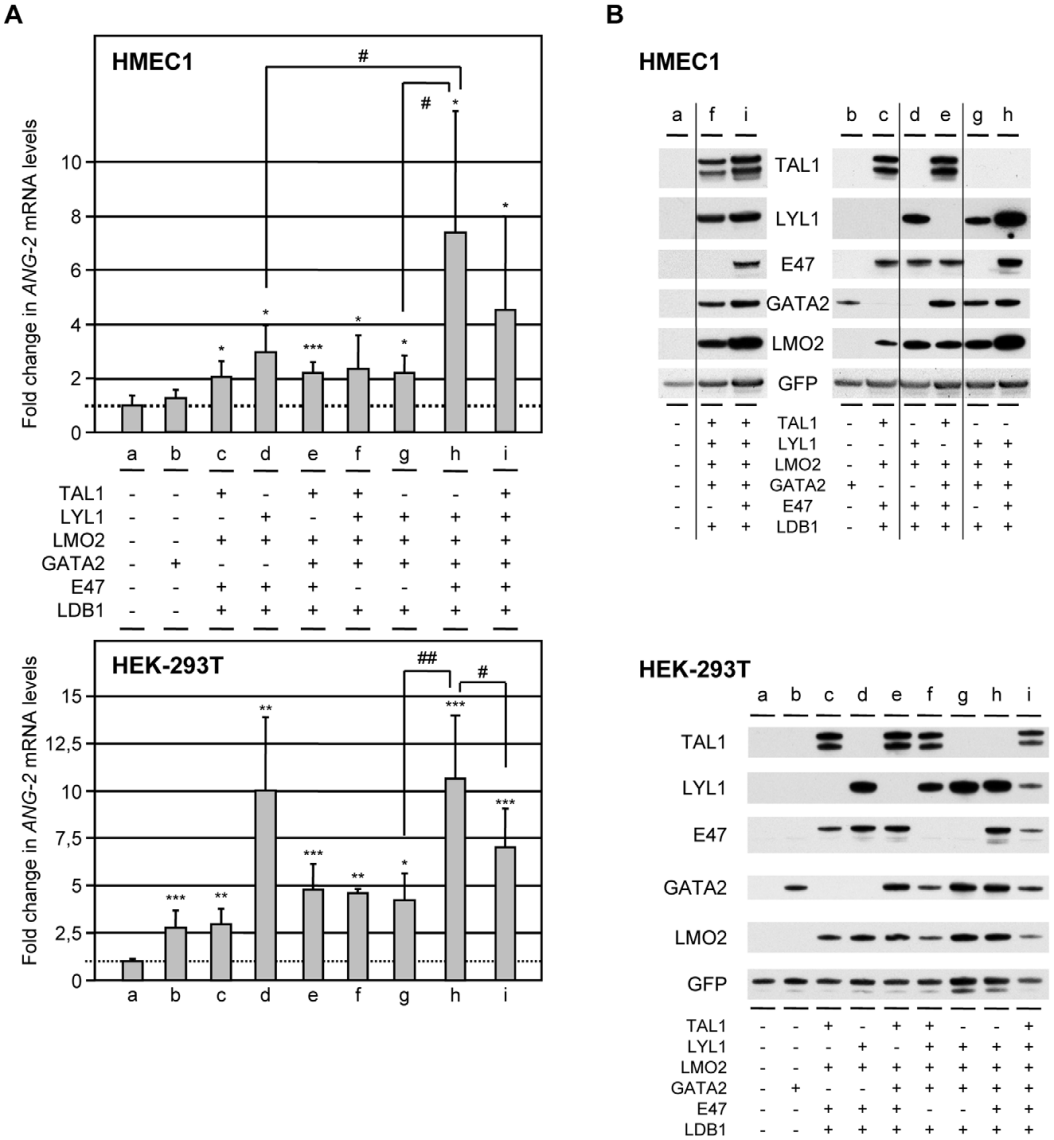
TAL1- and/or LYL1-containing complexes activate endogenous *ANG-2* transcription. Endothelial HMEC-1 cells or epithelial 293T cells were transfected with the indicated combination of expression plasmids along with pEGFP to control transfection efficiency; GFP-positive cells monitored by FACS analysis varied from 60 to 80% of total living cells. Total RNA and whole cell extracts (WCL) were prepared 48 h post-transfection for gene expression analysis. (A) *ANG2* mRNA expression was analyzed by RT-qPCR. The signals of mRNAs were normalized to those of *GAPDH*. Results are expressed as *ANG-2* mRNA levels relative to those of cells transfected with empty vector set at 1 (dotted line). Results are shown as the means ± SD of at least three separate experiments. Asterisks show significant differences with mRNA levels from cells transfected with empty vector. *or ^#^, *P*<0.05; ** or ^##^, *P*<0.01; ***, *P*<0.001. (B) WCL (10 µg) from the indicated transfected cells were analysed by immunoblot to control ectopic protein expression using the appropriate antibodies. Shown images are representative of a typical experiment; vertical lines indicate the suppression of non-informative lanes from a unique blot.

### Lentivirus production and transduction

Lentiviral particles were produced by co-transfection of 8.6 µg of pLKO.1-puro containing shRNA targeting TAL1, LMO2, LYL1 or a control shRNA (MISSION® shRNA – SIGMA-ALDRICH) together with two helper plasmids (8.6 µg of p8.91-Gag/Pol and 2.8 µg of pEnv-VSVG) in HEK-293T cells, using calcium phosphate precipitation. After 16 hours, cell culture medium was changed to fresh DMEM with 5% FBS. 48 hours later, pseudo particle-containing supernatants were centrifuged at 1.260 g for 10 min at 4°C and filtered on 0.45-µm nitrocellulose membrane, and stored in small aliquots at −80°C before use.

Endothelial cells plated in 10-cm dishes were transduced for 24 hours with 2 ml of virus-containing cell culture supernatants plus 1 ml of complete MV-2 medium containing 10 µg/ml polybrene. 72 hours after, transduced cells were selected in 1.3 mg/ml puromycine for 5 days and cultured thereafter in 1 mg/ml puromycine-containing complete medium.

**Figure 6:**
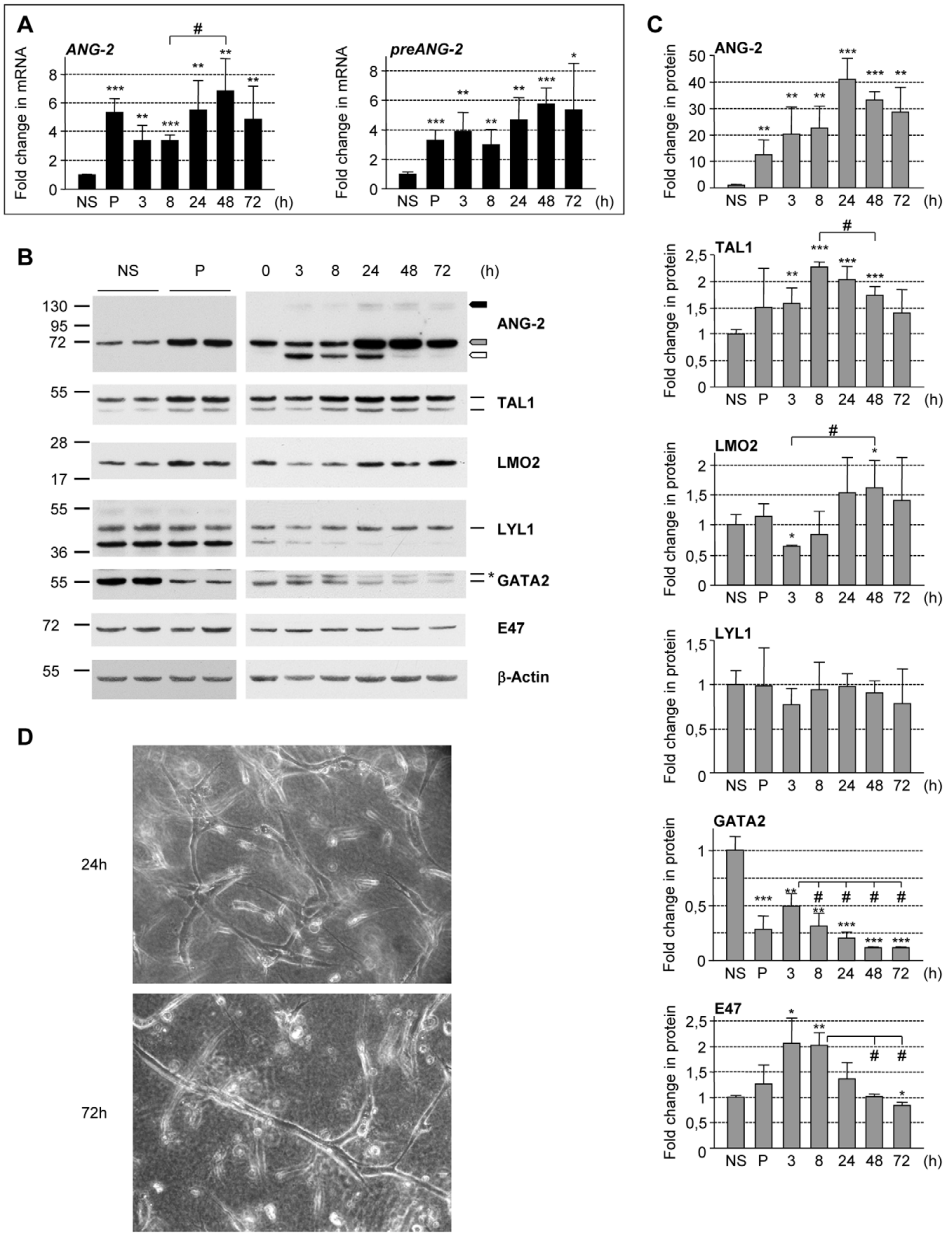
*ANG-2* gene activation during endothelial tubulogenesis coincides with the upregulation of TAL1 and LMO2 proteins. Confluent HUVECs were either maintained in basal endothelial medium containing 5% of fetal calf serum (NS: non-stimulated) or primed (P) with VEGF-A and bFGF for tubulogenesis. Primed HUVECs were seeded into collagen-I gels containing SDF-1α, SCF and IL3 to undergo vascular tube morphogenesis over 72 hours. Cells were recovered at the indicated time points to prepare total RNA and/or whole cell extracts. (**A**) Time-course analysis of *ANG-2* gene expression during *in vitro* tubulogenesis. *ANG-2* mRNA expression was analyzed by RT-q-PCR. To analyze *ANG-2* primary transcripts (*pre-ANG2*), *ANG-2* sequences from intron 1 were amplified. The signals of mRNAs were normalized to those of *GAPDH*. Data shown are RNA levels relative to the corresponding RNA levels of non-stimulated cells (NS) arbitrarily set at 1. Each bar is the mean +/− SD. of three independent experiments (*: P values versus NS) *, ^#^: *P*<0.05; **, ^##^: *P*<0.01; ***, ^###^: *P*<0.001. (**B, C**) Time-course analysis of ANG-2, TAL1, LMO2, LYL1, E47 and GATA2 protein expression during tubulogenesis. Whole cell lysates (30 µg) were used to analyze the indicated protein by immunoblot. (**B**): the images shown are representative of two tubulogenesis experiments. The arrows indicate the different forms of ANG-2: precursor (white), glycosylated monomers (grey); glycosylated dimers (black). The asterisk pinpoints the more slowly migrating isoform of GATA2, indicating post-translational modification. (**C**) Protein quantification was done by densitometric analysis. The values represent the amounts of the indicated protein normalized to β-Actin and relative to its amounts in non-stimulated cells, arbitrarily set at 1. Each bar is the mean +/− SD of two independent experiments (*: p values versus non-stimulated). *, ^#^: *P*<0.05; **: *P*<0.01; (**D**) Shown photographs illustrate the formation of the tubule network by primed HUVECs at the indicated time points of one experiment.

### Antibodies

The following antibodies (monoclonal: mAb; polyclonal: pAb) were used throughout this study: mouse anti-human TAL1 mAbs (clones 3BTL73 and 2TL136, [23]); goat anti-human TAL1 pAb (C-21) and rabbit anti-human E47 pAb (N-649) from Santa Cruz biotechnology, Inc; mouse anti-beta-ACTIN mAb (clone AC-15) and rabbit anti-Flag pAb were from Sigma-Aldrich; goat anti-human LMO2 pAb, mouse anti-human ANGIOPOIETIN-2 mAb, goat anti-human GATA2 pAb, all from R&D Systems (FRANCE). The rabbit anti-human LMO2 and anti-LYL1 pAbs were provided by Gerline Layh-Schmitt (USA) and Michael Cleary (USA) respectively.

**Figure 7:**
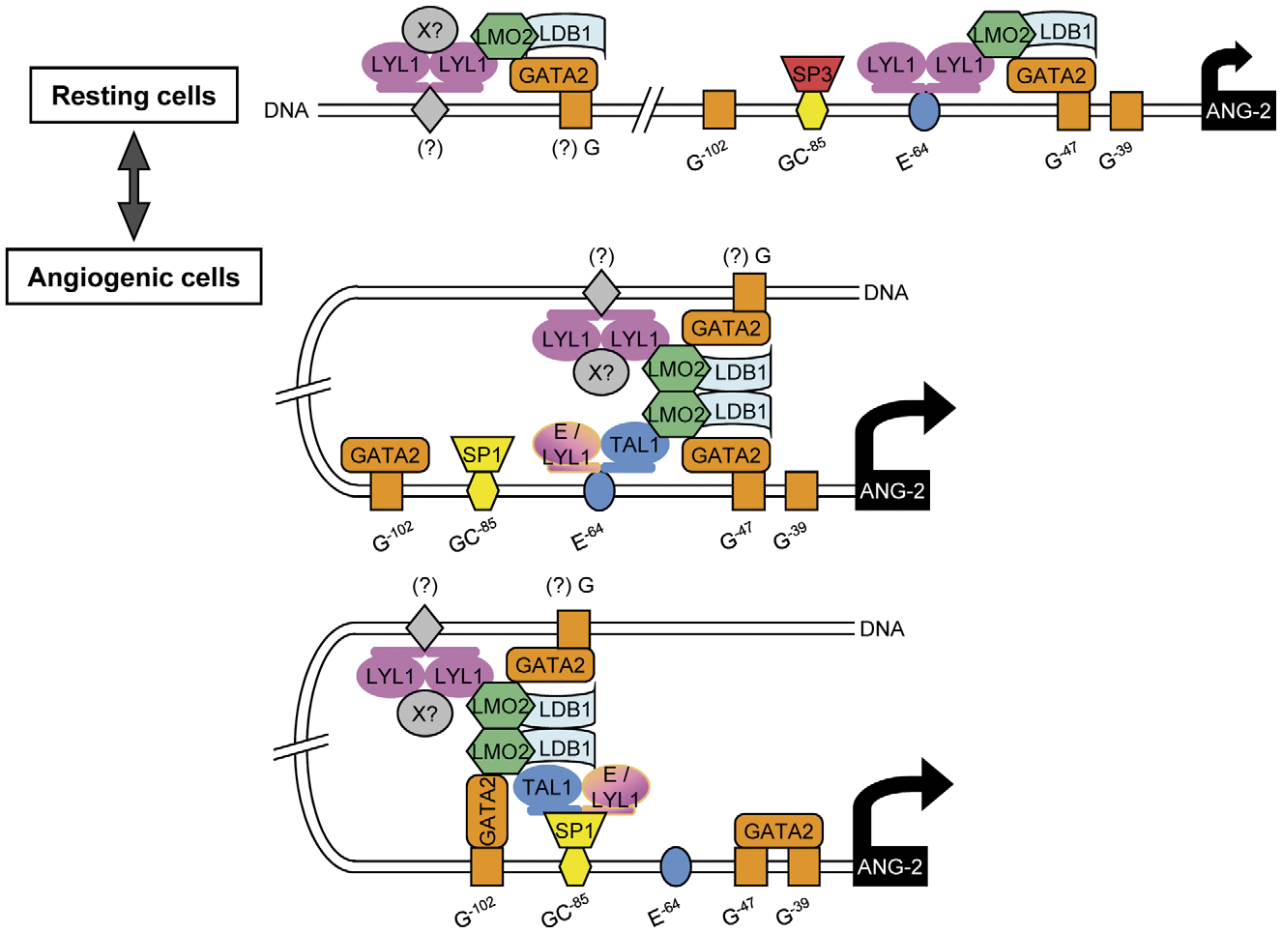
A proposed model illustrating how bHLHs may operate in endothelial cells to modulate *ANG-2* expression. The majority of HLH-dimers present in resting endothelial cells are LYL1 homodimers that, associated with LMO2 and GATA2 and with as yet unknown X cofactor(s), participate to constitutive *ANG-2* transcription. Upon angiogenic activation, the up-expression of TAL1 and LMO2 leads to the additional formation and binding of the classical TAL1-LMO2-containing complex onto the proximal *ANG-2* promoter through its interaction with GATA2 constitutively bound to the promoter, thereby enhancing its transcriptional activity. Higher-order complexes composed of two different bHLH dimers may also be formed due to the ability of LMO2 to dimerize through its interaction with the ubiquitous LDB1 nuclear protein.

### ANG-2 ELISA

ANG-2 protein levels in cell culture media were quantified by ELISA, using the Duoset Kit (R&D Systems, FRANCE), according to the manufacturer's protocol.

### Luciferase assays

#### Construction of the (−412, +73) *ANG-2* pGL3 reporters

The gene segment −412, +73 of human *ANG-2* promoter was amplified from genomic DNA by PCR using the following primers: forward: 5′-AACTCGAGAAACCGAAGTCCTGACCTATTTG-3′; reverse: 5′-AAAAGC TTGGAGGGCTGTGTCAGCTTTT-3′. PCR products were cloned in pGEM®-T Easy Vector (Promega) and sequenced. The 490 bp X*hoI*–H*indIII* fragment was then subcloned to the X*hoI* and H*indIII* sites of the pGL3 basic vector upstream of the *firefly luciferase* coding sequences (Promega). Mutations of the E-Box^−64^ in the promoter were performed using the Quick change II site-directed mutagenesis kit (Stratagen) using the following oligonucleotides (bold underlined characters indicated the mutated nucleotides):

forward: 5′-GGGCGGAGCAGCCCACTA**A**ACAT**A**TCTGGCTGCTC-3′
reverse: 5′-GAGCAGCCAGA**T**ATGT**T**TAGTGGGCTGCTCCGCCC-3′

Reporter plasmids were transfected using Jet PEI (OZYME, FRANCE) for HUVECs and HMEC-1 or using Promofectine (PromoCell GmBH, Heidelberg, GERMANY) for LLyECs, following manufacturer's instructions. The TK-RL plasmid encoding *renilla* luciferase was co-transfected with *firefly* luciferase reporters to correct for transfection efficiency. Normalized *firefly* luciferase activities were determined using the Dual-Glo luciferase kit (Promega, FRANCE).

### Chromatin immunoprecipitation (ChIP) assays

Chromatin immunoprecipitation assays were performed using exponentially growing LLyECs essentially as described [13]. Cleared chromatin corresponding to 10–15×10^6^ cells was incubated with specific antibodies directed against TAL1, LMO2, LYL1, GATA2, E47, acetylated-Histone H3 and species-matched control IgGs.

Aliquots of immunoprecipitated DNAs were analyzed by PCR with the following primers:

ANG-2 promoter Forward: 5′-TGGTTGGAGGGCAGGCATTC-3′
ANG-2 promoter Reverse: 5′-TTGGGAGGGCTGTGTCAGCTT-3′

Genomic untranscribed region upstream of the *cKIT* gene (−2295 to −2179) was amplified as a negative control with the following primers:

Forward primer: 5′-TCAAGGTTGGGCTGGGAGAT-3′
Reverse primer: 5′-GCTTATGTTCAGCCAGTACACACCA-3′

PCR products were analyzed by gel electrophoresis in the presence of Ethidium bromide. Fluorescence corresponding to each PCR product was analyzed using Image J software. Fold-enrichment of *ANG-2* target genomic regions immunoprecipitated by each specific antibody was normalized to the levels obtained with control IgGs and plotted as the increase over the level of enrichment at the negative control region.

### Co-immunoprecipitations assays in HEK-293T cells and immunoblot analysis

#### Plasmids used for transfections

The plasmids encoding TAL1, E47, LMO2, GATA2, LDB1 have been described previously [13]. Human *LYL1* cDNA tagged with Flag or HA was amplified by PCR and subcloned into pcDNA-3. The pEGFP-C1 plasmid is from Clontech. 293T cells (5×10^6^) were plated on 10-cm culture dishes 5 h prior to transfection.

293T cells were transfected by phosphate calcium precipitation with 7 µg (TAL1, LYL1, LMO2, GATA2, LDB1) or 1.4 µg (E47) or 2 µg (eGFP) of each expression vector (total amount of DNA per transfection was 42 µg). When necessary, the total amount of DNA was adjusted to 42 µg by adding empty vectors. After 48 h, cells were lysed on ice with the gentle lysis buffer (10 mM Tris-HCl, pH 7.5/150 mM NaCl/0.1% Triton X100/1 mM PMSF containing 2× complete EDTA-free protease inhibitor cocktail (Roche). Lysates were clarified by centrifugation at 14,000 rpm for 15 minutes at 4°C. Whole cell extracts (600 µg) were incubated with 2 µg of appropriate antibodies coupled to GammaBind G sepharose (GE-Healthcare) at 4°C for 4 hours; Flag-LYL1 and HA-tagged proteins were immunoprecipitated by adding anti-Flag-M2 affinity gel or anti-HA agarose (Sigma-Aldrich) respectively. Immunocomplexes were collected and washed four times with NET-2 buffer (50 mM Tris-HCl, pH 7.5/150 mM NaCl/0.05% Triton X100). The samples were boiled in western-loading buffer, resolved by SDS-PAGE and transferred onto Immobilon-P membranes (Millipore) for Western blot analysis. Western blot analysis was performed using the indicated antibodies and revealed with Luminata crescendo (Millipore).

### GST pull-down assays

Construction of pGEX-2T-TAL1 bHLH encoding GST fused to 172–289 residues of human TAL1 has been previously described [23]. The GST-LYL1 bHLH protein was produced from pGEX-2T in which GST sequences were fused to a 0.32 kb *AccI*-*SmaI* fragment from human *LYL1* cDNA encoding the residues 114–221 of LYL1 protein. The GST-E2A bHLH protein was produced from pGEX-3X in which GST sequences were fused to a 0.4 kb *MboI*-*BamHI* fragment from human *E2A* cDNA encoding the residues 283–397 of E47 protein. Bacterially expressed GST fusion proteins were adsorbed to glutathione-Sepharose beads and incubated with [^35^S]-Methionine-labeled *in vitro*-translated TAL1 or LYL1 proteins, and the bound proteins were analyzed by SDS-PAGE followed by autoradiography.

### Transient transfection of HMEC-1 cells and analysis of *ANG-2* gene activation

HMEC-1 cells were transfected using the Neon™ Transfection system (Life Technologies). The different combinations of transfected DNA contained 4 µg (TAL1, LYL1, LMO2, GATA2, LDB1) or 2 µg (E47, eGFP) of each expression vector; when necessary, the total amount of DNA was adjusted to 20 µg by adding empty expression vectors. Cells were prepared according manufacturer instructions in order to have one million of cells in 100 µl of Resuspension Buffer R. The cells were gently mixed with DNA and subjected to two 20 msec-pulses at 1400 V. Immediately after electroporation, cells were plated in MV2 medium containing 10% FCS in a 60 mm diameter dish. Five hours post-transfection, medium was changed in order to release dead cells. Cells were analyzed 48 h post-transfection.

### 3-D tubulogenesis in collagen-I gels

*In vitro* tubulogenesis assays were carried out according to the recently described two-steps model [32]. Briefly, proliferating HUVECs were primed overnight in complete MV2-medium containing 40 ng/ml of bFGF and VEGF-A (Peprotech, FRANCE). Primed cells were suspended into 2 mg/mL of collagen type I matrices containing 200 ng/mL of Stem-cell factor (SCF), stromal-derived factor-1 alpha (SDF-1α), and IL-3 (all from Peprotech, FRANCE). Cells were transferred into 24-well plate (3. 10^5^ cells per well) and allowed to undergo morphogenesis by incubation at 37°C in feeder medium containing reduced serum supplement and 40 ng/mL of bFGF as described [32]. At the indicated time points, gels were rinsed with PBS and incubated at 37°C with 150 µg/ml of high-purity Collagenase A (Sigma-Aldrich) and 2× complete EDTA-free protease inhibitor cocktail tablets (Roche Diagnostics GmbH, Germany). Cells were harvested after a 20 min-incubation to examine mRNA or protein cellular expression. Proteins were analyzed by electrophoresis onto NuPAGE 4–12% Bis-Tris gels (Invitrogen, FRANCE), followed by transfert onto immobilon membrane (Millipore).

### Gene mRNA expression analysis by real-time PCR

Total cell RNA was extracted using the High Pure RNA Isolation Kit (Roche, FRANCE) following the manufacturer's instructions. RNAs were primed with oligo(dT) or with random primer and reverse transcribed with SuperScript II (Invitrogen, FRANCE). The sequences of primers used to amplify the different cDNAs were the followings:

*GAPDH –* Forward 5′-ACACCCACTCCTCCACCTTT

*GAPDH –* Reverse 5′-TCCACCACCCTGTTGCTGTA

*LYL1-* Forward 5′- CATCTTCCCTAGCAGCCGGTTG

*LYL1-* Reverse 5′- GTTGGTGAACACGCGCCG

*ANG2*- Forward 5′-TCAGGGCCCTACCACTAAGGGCTTGCC

*ANG2*- Reverse 5′- TGGGACGCTTGGGAACTGTGCCCCT

pre-*ANG2*-Forward 5′- TCAGGGCCCTACCACTAAGGGCTTGCC

pre-*ANG2*-Reverse 5′- TGGGACGCTTGGGAACTGTGCCCCT

### Statistical analysis

All results were expressed as the means +/− standard deviation (S.D.). Significance of differences was determined with the Student *t* test with significance at *P*<0.05.

## Results

Small interfering RNAs (siRNA) were used to reduce the levels of endogenous TAL1, LYL1 or LMO2 protein in two different human primary endothelial cell types: HUVECs derived from umbilical vein and CB-ECs purified from umbilical cord blood. Preliminary genome-wide array analysis comparison of TAL1-, LYL1- or LMO2-silenced cells versus siRNA control-treated cells identified several hundred of gene changes (greater than 2-fold) as a result of silencing of either factor (not shown). Among the genes that were similarly modulated by TAL1-, LYL1- and LMO2-knockdown in both HUVECs and CB-ECs, Angiopoietin-2 here after named ANG-2, was further investigated since it fulfilled our criteria: ANG-2 is almost exclusively expressed by endothelial cells and it plays a major role in adult angiogenesis and lymphangiogenesis (see review [33]).

### TAL1-, LMO2-, or LYL1-depletion down-regulates *Angiopoietin-2* expression

We validated down-regulation of *ANG-2* in *TAL1-*, *LMO2*- or *LYL1*-depleted HUVECs, by quantitative RT-PCR and immunoblot analysis (Fig. 1A). *ANG-2* mRNA levels were significantly reduced in *TAL1*-, *LYL1*- and *LMO2*-depleted cells, by 65%, 81% and 54% respectively, as compared to *siGFP-*treated ECs. Consequently, the silencing of any of the three factors caused a dramatic decrease in intracellular ANG-2 protein amounts.

Given the key role of ANG-2 in lymphangiogenesis, similar experiments were conducted with lymphatic ECs derived from human lung micro capillaries (LLyECs). Long-term silencing mediated by lentiviruses encoding shRNA targeting *TAL1*, *LYL1* or *LMO2* also dramatically reduced *ANG-2* expression by 86%, 92% and 76% respectively (Fig. 1B). Consequently, ANG-2 protein was no more detectable in whole cell extracts of the three depleted cell populations.

ANG-2, stored in specialized intracellular compartments, the Weibel Palade Bodies (WPBs) in resting ECs, is constitutively released in proliferating ECs and rapidly exported upon activation [34]. ANG-2 constitutive secretion, as measured by ELISA, was significantly reduced in TAL1-, LYL1- and LMO2-depleted HUVECs, by 54%, 50% and 42%, respectively (Fig. 1C). Constitutive ANG-2 secretion by LLyECs was more than 2-fold higher than by HUVECs, as reported [35]. ANG-2 release by shTAL1-, shLYL1- and shLMO2-transduced LLyECs was also dramatically reduced, by 82%, 75% and 80% respectively relative to control cells.

Collectively, these data confirmed that the depletion of TAL1, LYL1 or LMO2 down-regulates *ANG-2* expression at both the mRNA and protein levels.

### TAL1, LYL1, LMO2, and GATA2 bind the *ANG-2* proximal promoter at a conserved region containing an E-Box-GATA composite element

We surveyed the *ANG-2* locus for the occurrence of evolutionarily conserved sequences and found that highly conserved sequences were limited to the promoter region, reported to confer endothelial-specific *ANG-2* expression [36]. This region contains 3 conserved GATA sites, one E box and one SP1/SP3 site (Fig. 2A). Of note, the E-box^−64^, matching the preferred sequences bound by TAL1-E47 and LYL1-E47 heterodimers [37], [38], is separated by 11 nucleotides from the GATA^−47^. This particular E-box-GATA arrangement is found in the promoter or regulatory regions of genes regulated by TAL1-containing complexes in blood and endothelial cells [7], [13].

To investigate the role of these conserved elements, we cloned a 490 bp fragment of human *ANG-2* containing 412 bp upstream of the transcription start site and 73 bp untranslated 5′ region upstream in a Luciferase reporter plasmid. To assess the significance of the E-box^−64^, we disrupted this motif in the context of the reporter construct and tested wild type and mutated promoter activity in transient transfections in ECs (Fig. 2B). The mutated promoter showed reduced activity compared to the wild-type promoter in HUVECs, LLyECs and HMEC-1 cells down to 52%, 75% and 60% respectively. These experiments demonstrated that the E^−64^ element is required for full endothelial-specific activity of the *ANG-2* promoter.

Our above observations were consistent with the possibility that TAL1, LYL1, LMO2 and GATA2 operate together on the *ANG-2* promoter as they do in immature hematopoietic cells. Therefore, we performed chromatin immunoprecipitation (ChIP) assays with antibodies against TAL1, LYL1, LMO2, GATA2 and E47 in LLyECs, since they expressed *ANG-2* mRNAs at higher levels than HUVECs. As shown in Figure 2C, TAL1, LYL1, LMO2, and GATA2 were found to bind significantly the *ANG-2* promoter region containing these sequences in LLyECs.

### Formation of TAL1- and/or LYL1- containing complexes in HEK-293T cells

We next investigated whether TAL1 and LYL1 function in the same complex within the cells. Human embryonic kidney 293T cells were transfected with expression vectors encoding TAL1 and LYL1 carrying a Flag epitope tag, either separately or in combination with their common partners E47, LMO2 carrying a HA epitope tag and GATA2, along with pEGFP as an internal control. GFP-positive cells monitored by FACS analysis varied from 70 to 85% of total living cells (data not shown). Immunoblot analysis of cellular protein contents showed variations in the levels of LYL1 and LMO2 in the different combinations (Fig. 3A and 3C, input). These differences in protein levels were not due to variations in *LMO2* and *LYL1* mRNA levels, as they were found by RT-qPCR to be similar in transfected cells with the different combinations (data not shown), in agreement with comparable GFP expression. This allowed us to make several observations from the analysis of cellular protein contents.

TAL1 levels were not affected by the co-expression of other components, however a shift of TAL1 was observed upon the addition of its partner E47 (compare lanes b, d, e with lanes a, c). Phosphatase treatment showed that this shift is due to TAL1 hyperphosphorylation mediated by E47 (Fig. 3B). In contrast, LYL1 protein levels were strongly impacted by the co-expression of LMO2 (compare lanes a, c, d, f with g), which was itself dependent on the presence of its partner GATA2 (compare b and d). Of note, LYL1 and LMO2 levels displayed similar modulations and were strongly reduced when co-expressed with TAL1 and E47 (compare a to b and c to d). These data indicate that variations in LYL1 and LMO2 protein levels result from post-translational events, and suggest that LYL1 and LMO2 may function in cells as mutually stabilizing factors.

Co-immunoprecipitations (IPs) were carried out using equal amounts of whole cell extracts (WCE) using α-TAL1 antibody, α-Flag antibody to pull-down LYL1 (Fig. 3A) or α-HA to precipitate LMO2 (Fig. 3C). The amounts of immunoprecipitated TAL1, Flag-LYL1 and HA-LMO2 with their respective antibody reproduced the variations of the proteins observed in WCE (input), validating the three antibodies for IP.

In keeping with their known interactions with TAL1, both E47 and LMO2 were efficiently brought down with α-TAL1 mAb. Of note, equal amounts of LMO2 were observed in each IP, irrelatively to the LMO2 quantity present in WCE (see lanes b and c, input and IP α-TAL1). The amounts of LYL1 co-precipitating with the α-TAL1 antibody paralleled the levels of LYL1 protein in input. Similarly, in the reciprocal LYL1-IP using α-flag antibody, the levels of co-immunoprecipitated TAL1 followed those of LYL1 in WCE; notably, the highest amounts of TAL1 were observed in the combination that includes LMO2 and GATA2 but lacks E47 (Fig. 3A, lane c α-Flag IPs). In contrast to TAL1, LYL1 poorly associated with E47 (lanes b-d, α-Flag IPs). The LMO2-IPs using the α-HA antibody confirmed strong LMO2-TAL1 interaction that was stimulated upon the addition of E47 (Fig. 3C, compare a and b), in keeping with the preferred LMO2-association with TAL1 heterodimers [39]; in addition, LMO2 nicely interacted with LYL1 in the absence of E47 (Fig. 3C, compare a to b, and c to d). Together these data indicate that TAL1 and LYL1 are present in the same complexes, which also include LMO2 and GATA2.

These data prompted us to address whether TAL1 and LYL1 could interact directly through their HLH domain. Therefore, *in vitro* binding assays were performed using *in vitro*-translated TAL1 and LYL1 and recombinant GST-proteins containing the bHLH domains of E47, TAL1 and LYL1 (Fig. 4A). As expected, TAL1 strongly interacted with E47 but not with itself. Whereas no specific direct interaction between TAL1 and LYL1 or LYL1 and E47 was detected in this assay, LYL1 was found to homodimerize *in vitro*.

To ascertain whether LYL1 also homodimerizes in cells, co-IPs were performed in 293T cells cotransfected with two LYL1 constructs carrying a Flag epitope tag or a HA epitope tag (Fig. 4B). When Flag-LYL1 was immunoprecipitated with α-Flag, HA-LYL1 was co-immunoprecipitated. In the reciprocal coIP using α-HA, Flag-LYL1 was brought down with HA-LYL1. Importantly the co-expression of LMO2 and GATA2 markedly increased the amounts of LYL1 homodimers in cell extracts. Collectively, these data indicate that LYL1 poorly interacts *in vivo* with E47, but rather forms homodimers as well as heterodimers with TAL1 that are both facilitated by LMO2 and stabilized in the presence of GATA2.

### Several combinations of ectopic TAL1, LYL1, LMO2 and GATA2 activate endogenous *ANG-2* expression

We next assessed whether TAL1 and/or LYL1 and their cofactors directly activate *ANG-2* transcription. HMEC-1 endothelial cells were transfected with vectors encoding TAL1 and/or LYL1, in combination with their partners E47, LMO2 and the two LMO2-cofactors, GATA2 and the ubiquitous LIM-domain-binding protein1 or LDB1, along with peGFP as an internal control. In agreement with similar GFP expression, the analysis of the expression of the different transgenes by immunoblot (Fig. 5B) did not reveal major variations in LYL1 and LMO2 protein levels in HMEC1 cells transfected with the different vector combinations. This is likely to be due to the presence of both GATA2 and LDB1, known to stabilize LMO2 that in turn prevents LYL1 degradation (see our above data).

*ANG-2* mRNA levels were determined by RT-q-PCR (Fig. 5A). Whereas GATA2 alone did not modify endogenous *ANG-2* mRNA levels (lane b), any combination that could lead to the formation of TAL1-E47 (lanes c, e), TAL1-LYL1 (lane f) or LYL1-E47 (lane d) heterodimers elicited a similar *ANG-2* stimulation (about 2-fold). LYL1 as the sole bHLH (lane g) was also able to stimulate *ANG-2* in the absence of E47, consistent with its ability to homodimerize (our above data). Maximal *ANG-2* stimulation was observed when LYL1 was combined with E47, LMO2 and its cofactors LDB1 (3-fold, lane d) and GATA2 (7.4-fold, lane h) that might generate both LYL1 homodimers and LYL1-E47 heterodimers.

The same combinations of expression vectors were tested in the non-endothelial 293T cells that do not normally express *ANG-2*. In agreement with our data in HMEC-1 cells, any combination tested was able to turn on *ANG-2* transcription in 293T cells (Fig. 5A). Importantly, the sole expression of GATA2 was sufficient to switch on *ANG-2* transcription (2.6-fold, lane b), and the addition of any HLH-dimer triggered a further increase of *ANG-2* activation (∼5-fold, lanes c, e-g). Again, *ANG-2* stimulation was maximal with the concurrent activity of LYL1 homodimers and heterodimers (up to 10-fold, lanes d, h).

Collectively, these data demonstrated that several complexes including TAL1 and/or LYL1 and their partners LMO2 and GATA2 have the ability to stimulate *ANG-2* expression in ECs as well as in non-endothelial cells.

### *ANG-2* activation in angiogenic ECs coincides with TAL1 and LMO2 up-regulation

We next investigated the expression of the different components of the LMO2-complexes that may contribute to *ANG-2* regulation in resting conditions (i. e. in non-stimulated ECs) and during angiogenesis, using a recently described two-step model that recapitulates physiological vascular tube morphogenesis and sprouting [32], [40]. HUVECs were primed overnight with two pro-angiogenic cytokines (VEGF and bFGF) and thereafter were exposed to hematopoietic cytokines (SCF, IL3 and SDF-1α) into 3D-collagen matrices. The cultures were allowed to assemble into tubular network over 3 days and *ANG2* mRNA and protein expression analysis was performed at different time-points of the process (Fig. 6).

As expected, *ANG-2* mRNA levels were low in non-stimulated ECs and markedly increased (about 5-fold) upon activation with the pro-angiogenic cytokines; *ANG-2* mRNA levels were slightly reduced in the first hours of 3-D tubulogenesis and increased subsequently to reach a maximum at 48 h (Fig. 6A, left). Primary *ANG-2* transcript analysis indicated this augmentation throughout the process was not due to mRNA stabilization but rather to transcriptional activation (Fig. 6A, right). Intracellular ANG-2 protein contents (Fig. 6B & 6C) dramatically increased upon angiogenic and throughout tubule-forming process, reaching its maximum (40x-fold compared to NS cells) at 24 h. Neo-synthesis of ANG-2, as visualized by the presence of ANG-2 precursor, correlated with *ANG-2* transcription leading to the gradual accumulation of the glycosylated mature forms of ANG-2, once the tubules were formed.

All transcriptional regulators tested were present in both non-stimulated and activated ECs, but exhibited distinct modulation (Fig. 6B & 6C). TAL1 protein, up regulated upon priming, gradually increased during tubule formation reaching a 2.3-fold peak between 8 h and 24 h. LMO2 protein was transiently reduced in early 3D-angiogenesis, then gradually increased and remained at high levels in formed tubules. LYL1 exhibited no change throughout the process. GATA2 protein amounts, high in non-stimulated ECs, strongly decreased (around 70%) upon priming. Within the first hours of tubulogenesis, we observed a 1.8-fold increase in GATA2 levels with concurrent production of a slower migrating GATA2 isoform, which was present until the end of the process. A further decrease in GATA2 occurred in the later steps of tubulogenesis. A transient 2-fold increase in E47 was observed in early steps of tubulogenesis.

Altogether these data show that activation of *ANG-2* in early endothelial morphogenesis correlates with the up-expression of TAL1 and LMO2. In newly formed endothelial tubes, *ANG-2* transcription appears to be essentially mediated by LYL1-LMO2-complexes.

## Discussion

This study was to further explore the role of three well-known hematopoietic factors TAL1, LMO2 and LYL1 in endothelial cells. Here, we identified *ANG-2* that encodes a major regulator of angiogenesis as an endothelial target of the 3 factors. The angiopoietins ANG-1 and ANG-2 and their ligand TIE2 play a central role in regulating vascular endothelium functions. ANG-1 acts as Tie2 agonist to promote and maintain mature blood vessels. ANG-2, stored in WPBs of ECs, is released upon activation and acts as ANG-1 antagonist in an autocrine manner. ANG-2 primes and activates endothelium to respond to other angiogenic factors and destabilizes vessel coverage by pericytes, an essential step to initiate angiogenesis (reviewed in [33]).

Given its essential function in the vascular system, *ANG-2* must be tightly controlled through a complex interplay between positive and negative regulators. ETS proteins regulate *ANG-2* transcription through ETS-binding sites at the *ANG-2* promoter [41], and in response to hypoxia, HIF-1α activates *ANG-2* through binding to a HRE within the first intron [36], [42]. Upon stress, FOXO1b rapidly induces *ANG-2* expression after inhibition of the PI3K/AKT pathway [43], and high glucose treatment of ECs induces *ANG-2* activation through the binding of SP1 to the GC-box^−85^ of the *ANG-2* promoter, which takes the place of the repressor SP3 [44]. Here, we identify TAL1, LYL1 and LMO2 as new pivotal participants in the complex network of *ANG-2* transcriptional regulators.

Depletion of any of the 3 factors in proliferating ECs down-regulates *ANG-2* expression and we found that TAL1, LYL1, LMO2 and its partner GATA2 bind the *ANG-2* promoter in a conserved region that is characterized by the presence of an Ebox-GATA element. Although the sequence of the E-box-^64^ matches the preferred consensus E-Box for TAL1/E and LYL1/E dimers, its mutation caused a moderate reduction of the promoter, presumably due to other important intact elements in the promoter (Fig. 2). Several studies have shown that direct DNA-binding of TAL1 is dispensable for specification of hematopoiesis [45] and that TAL1 can activate transcription in the absence of an E-box through its association with other transcription factors [7]. In immature hematopoietic cells, the presence of an SP1-motif close to an Ebox-GATA element is significantly associated with GATA2-TAL1 occupancy of this element and a GATA-motif is a better predictor of TAL1 occupancy than is an E-box [46], [47]. In our study, whereas GATA2 and TAL1- or LYL1-dimers additively activated *ANG-2* transcription in non-endothelial cells, ectopic GATA2 alone did not stimulate *ANG-2* transcription in HMEC-1 endothelial cells, in keeping with GATA2 acting as a constitutive endothelial *ANG-2* activator [36]. Hence, we assume that a part of TAL1- and LYL1-complexes are tethered at the *ANG-2* promoter through GATA2 and/or SP1 bound respectively to the GATA-motifs and the GC-box^−85^.

Important variations in GATA2 protein levels between resting and angiogenic ECs were observed (Fig. 6B). High expression of GATA2 is present in non-stimulated HUVECs, whereas a strong reduction in GATA2 occurred upon angiogenic cytokines stimulation. Importantly, in the early steps of tubule formation, the transient GATA2 up-regulation is associated with the presence of an additional slower migrating isoform that is maintained throughout the process. Of note, the production of this isoform presumably by post-translational modification such as phosphorylation, is concomitant with *ANG-2* up-regulation.

In two independent genome-wide occupancy analyses, GATA2 was found to bind regulatory elements of several genes that confer endothelial specificity including *ANG-2* [48], [49]. Hence, high GATA2 expression observed in non-stimulated ECs might be responsible for the constitutive expression of these genes. Upon stimulation with hematopoietic cytokines including IL-3, GATA2 phosphorylation through the activation of the ERK pathway, (reviewed in [50]) may modulate the binding site preferences and/or enhance protein interactions notably with LMO2 and TAL1, leading to further activation of the *ANG-2* locus. Our findings support a model in which TAL1 and LYL1 function in relay with LMO2 and GATA2 to modulate *ANG-2* in endothelial cells (Fig. 7). In resting ECs, where TAL1 is absent, the LMO2-LYL1 complexes present at the *ANG-2* promoter with GATA2 and/or with as yet unknown cofactors (indicated as X in the figure) may elicit low constitutive *ANG-2* expression. Upon angiogenic activation, the concomitant up-regulation of TAL1 and LMO2 produces additional complexes that are recruited by phosphorylated GATA2 and/or SP1 at the *ANG-2* promoter to further stimulate its transcriptional activity. Several bHLH-complexes may coexist in proliferating EC population, however each cell presumably contains only one type of complex according to the respective concentration of the different bHLHs. Given the ability of LMO2 to dimerize through its interaction with LDB1 [51], higher-order complexes composed of two different bHLH dimers may also be formed.

Endothelial LYL1 activity is involved in maturing newly formed blood vessels, notably by regulating the expression of proteins, which mediate the adhesion and stabilization of vessels to extracellular matrix [26]. Here, we found that LYL1 also controls the expression of ANG-2, a vascular destabilizing factor. Kinetics analysis of ANG-2 protein expression during tubulogenesis relieves this apparent discrepancy (Fig. 6). Indeed, ANG-2, produced throughout the process, gradually accumulates within the cells as mature glycosylated ANG-2 proteins, which are stored in the WPBs [34]. At the end of the process, LYL1 and LMO2 remain at high levels, in agreement with their co-expression in resting endothelium, where TAL1 is absent. Hence, we propose that LMO2 and LYL1 act together in maturing and resting vessels to replenish the intracellular pool of ANG-2. It will be interesting to investigate whether they are also involved into the mechanisms whereby ANG-2 is stored in WPBs.

Consistent with the highest expression of TAL1 and LMO2 is observed at sites of vascular remodeling, the classical TAL1-LMO2 complex regulates important actors of endothelial morphogenesis. It controls the expression of the VEGF-receptor-2 during early vascular development [14] and in adult, it activates *VE-cadherin* [13], encoding the major component of endothelial adherens junctions that plays a crucial role in vascular morphogenesis [52]. We now identify *ANG-2* as a new component of the genetic program controlled by TAL1-LMO2-complex. In agreement, the highly angiogenic tumors developed in *Lyl1*-deficient mice exhibit sustained TAL1 expression with a concurrent up-expression of *VE-Cadherin* and *ANG-2*, while TAL1 was not detected in tumor vessels from wild-type mice [26].

The identification of TAL1, LMO2 and LYL1 as new critical regulators of ANG-2 expression definitively establishes the important role of these factors in endothelial cells, and more particularly in tumor angiogenesis and development. ANG-2 up-regulated in tumors mainly by ECs, stimulates tumor angiogenesis as well as metastatic dissemination. Tumor-derived ANG-2 also recruits Tie2-expressing monocytes and stimulates their proangiogenic functions, thereby stimulating tumor growth and facilitating metastatic dissemination [53]. The development of new therapeutics molecules targeting the ANG-2-Tie2 axis is promising since, they may not only inhibit tumor angiogenesis directly but also indirectly reduced metastasis [54], . Hence, the endothelial TAL1-LMO2 activity may represent a novel potential target for inhibiting ANG-2 up-regulation in developing tumors.
